# Exposing the grey seal as a major predator of harbour porpoises

**DOI:** 10.1098/rspb.2014.2429

**Published:** 2015-01-07

**Authors:** Mardik F. Leopold, Lineke Begeman, Judith D. L. van Bleijswijk, Lonneke L. IJsseldijk, Harry J. Witte, Andrea Gröne

**Affiliations:** 1 Department of Ecosystems, Wageningen IMARES, PO Box 167, 1790 AD, Den Burg, Texel, The Netherlands; 2 Department of Pathobiology, Faculty of Veterinary Medicine, Utrecht University, Yalelaan 1, 3584 CL, Utrecht, The Netherlands; 3 Molecular Biology Laboratory, Department of Biological Oceanography, Royal Netherlands Institute for Sea Research, PO Box 59, 1790 AB, Den Burg, The Netherlands

**Keywords:** marine mammals, mutilation, predation, DNA, bite mark, decision tree

## Abstract

Harbour porpoises (*Phocoena phocoena*) stranding in large numbers around the southern North Sea with fatal, sharp-edged mutilations have spurred controversy among scientists, the fishing industry and conservationists, whose views about the likely cause differ. The recent detection of grey seal (*Halichoerus grypus*) DNA in bite marks on three mutilated harbour porpoises, as well as direct observations of grey seal attacks on porpoises, have identified this seal species as a probable cause. Bite mark characteristics were assessed in a retrospective analysis of photographs of dead harbour porpoises that stranded between 2003 and 2013 (*n* = 1081) on the Dutch coastline. There were 271 animals that were sufficiently fresh to allow macroscopic assessment of grey seal-associated wounds with certainty. In 25% of these, bite and claw marks were identified that were consistent with the marks found on animals that had tested positive for grey seal DNA. Affected animals were mostly healthy juveniles that had a thick blubber layer and had recently fed. We conclude that the majority of the mutilated harbour porpoises were victims of grey seal attacks and that predation by this species is one of the main causes of death in harbour porpoises in The Netherlands. We provide a decision tree that will help in the identification of future cases of grey seal predation on porpoises.

## 1. Introduction

Marine mammals strand occasionally with large, fatal wounds. Suggested causes include ducted propellers [1], fishermen confronted with by-catch [2], and predators or scavengers [3–5]. Over the past decade, hundreds of severely mutilated harbour porpoises (*Phocoena phocoena*) have been found along the southeastern North Sea coastline [6], the cause of the wounding being unknown. This has resulted in controversy among scientists, the fishing industry and conservationists as to whether such mutilations were anthropogenic in origin or naturally inflicted by predators.

Research on predated livestock and protected wildlife species has demonstrated that the presence of salivary DNA of predators in bite wounds can be used to specifically identify the predator species [7–9]. Acute haemorrhages in the bite wounds and other lesions found during autopsy aid evaluation of the cause of death, and help distinguish between predation of a live animal and post-mortem scavenging. DNA degradation and/or the flushing out of predator saliva occurs quickly in bodies submerged in water [10], and therefore, in mutilated marine mammals, the predator's DNA is most likely to be demonstrated in victims that are found fresh after having died rapidly from the wounds. As there is frequently a long interval between death and autopsy of stranded marine mammals, diagnosis of a predator attack by DNA is difficult. Despite this, grey seal (*Halichoerus grypus*) DNA has recently been demonstrated within bite wounds on mutilated harbour porpoises [11].

The aims of this study were to evaluate the characteristics and incidence of grey seal-associated wounds found on harbour porpoises stranded along the Dutch coastline, determine criteria to establish if these were made ante- or post-mortem, and develop a decision tree to help investigators undertaking autopsies of small cetaceans to identify interactions with grey seals accurately. We show that a substantial proportion of harbour porpoises that stranded on the Dutch coast were mutilated by grey seals. We also conclude that most cases involved active killing and that only a small proportion can be attributed to post-mortem scavenging. This makes predation by grey seals one of the main causes of death in harbour porpoises currently stranding in The Netherlands.

## 2. Material and methods

### (a) Porpoises used for characterization of grey seal-associated wounds

**Figure 1.**
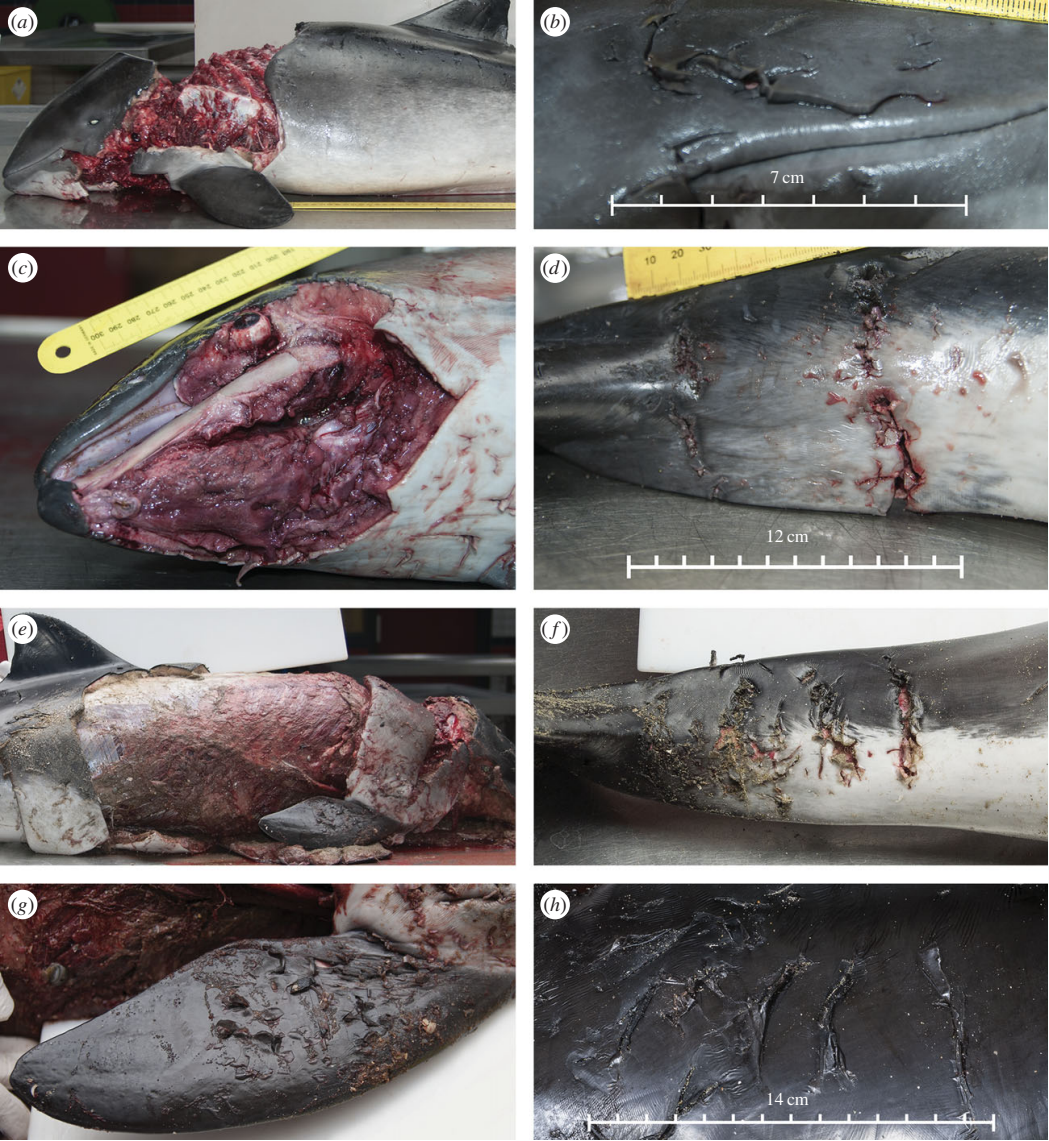
Macroscopic photographs of the harbour porpoises with grey seal DNA-positive wounds. (*a*) Pp 3, left side shows absence of large pieces of skin, blubber and musculature. (*b*) Pp 3, right side of the maxilla showing repetitive puncture lesions on the head (‘head mark’). (*c*) Pp 1, absence of large amounts of skin and blubber in the mandible and throat area, leaving the fractured mandibular bone bare. (*d*) Pp 1, two lines of parallel running puncture lesions on the tailstock, the lesions were bilateral symmetrical (not visible in picture) (‘tailstock mark’). (*e*) Pp 2, large skin and blubber defects on the body wall leaving ribs and musculature bare. (*f*) Pp 2, repetitive bite marks on the tailstock similar to Pp 1, figure 1*d*. (*g*) Pp 2, flipper with repetitive punctures on the dorsal surface that were mirrored on the palmar surface (not visible in picture) (‘flipper punctures’). (*h*) Pp 2, five parallel running scratches on the left lateral body wall (‘scratches’).

Grey seal DNA was demonstrated in various bite marks on three mutilated harbour porpoises [11]. These wounds showed macroscopic and microscopic acute haemorrhages, indicating that these lesions had been inflicted during life, just prior to death. Figure 1 shows the lesions that were present on these animals and table 1 shows which lesions were swabbed and which lesions were positive for grey seal DNA. All three animals were in good nutritional condition and had fed shortly prior to death, as shown by the presence of partly digested prey in their stomachs. The mutilations were considered fatal and exsanguination was the most likely cause of death.

**Table 1.** Wounds presence and number of swabs tested in three mutilated harbour porpoises (Pp 1–3); numbers give swabs taken/swabs that tested positive for grey seal DNA; n-s = lesion present but not swabbed; abs = lesion absent.

wound	Pp 1	Pp 2	Pp 3
blubber defect (edge)	n-s	1/0	2/0
tailstock punctures	1/1	2/2	abs
head punctures	1/0	n-s	1/1
flipper punctures	n-s	n-s	n-s
parallel scratches	n-s	n-s	abs

### (b) The incidence of grey seal bite marks

The incidence of grey seal attacks on harbour porpoises was determined with a retrospective study of 1081 harbour porpoises that stranded on the Dutch coastline and were autopsied between 2003 and 2013. Porpoises were collected on the basis of available local logistics, irrespective of the preservation of the carcass. All carcasses had been photographed, paying special attention to any skin and blubber lesions. We used these photographs to assess the presence or absence of lesions associated with grey seal interactions. When the preservation state of the carcass, the absence of body part, or the quality of the pictures made assessment impossible, cases were scored as ‘unknown’.

### (c) Distinguishing ante-mortem grey seal-associated wounds from post-mortem scavenging

For each suspected grey seal mutilation case, the autopsy report was reviewed. Criteria used to denote an attack rather than post-mortem scavenging by a grey seal were: no definitive other cause of death (e.g. infectious disease or emaciation), presence of macroscopic or microscopic acute haemorrhages associated with the presumed bite marks, a good nutritive condition (see below) and evidence that the porpoise had fed shortly prior to death (i.e. prey remains in the stomach).

### (d) Nutritional condition code

For each porpoise, the nutritional condition code (NCC) was scored on a scale from 1 (very fat and muscular) to 6 (emaciated) [2]. The relationship between NCC and the probability of the presence of grey seal-associated interaction was analysed by generalized linear modelling (including a binomial error distribution and logit link) in which we used the ordered categorical variable NCC as a continuous variable. To test whether NCC could be used as a continuous variable, we first fitted a generalized additive model (GAM) to see if there was a nonlinear pattern between the probability of predation and the NCC status. A nonlinear pattern would suggest that the different levels of NCC have different lengths (e.g. from NCC1 to NCC2 is not the same as the distance between NCC 2 and 3). The GAM showed that the relationship was strictly linear (electronic supplementary material, figure S1), confirming that NCC can be used as a continuous variable, and 95% confidence limits were determined using a simulation [12]. Porpoises have a thicker blubber layer in winter [13], and this seasonal effect is likely to be reflected in the NCC. As probable grey seal victims were more commonly found in winter (electronic supplementary material, figure S2), we restricted this analysis to those porpoises found stranded from December up to and including March, to remove this seasonal effect.

## 3. Results

Three harbour porpoises (figure 1*a*,*c*,*e*) were examined. Wounds that contained grey seal DNA were small, repetitive incisions present on the head (figure 1*b*) or bilaterally on the tailstock (figure 1*d*,*f*). In addition, presumed grey seal bite marks were present on the flippers (figure 1*g*) and presumed grey seal nail rake marks [4] were present as five parallel scratches on the bodies of the DNA-positive porpoises (figure 1*h*). Large, presumably fatal defects in the epidermis (which extended through the full thickness of the blubber, with substantial parts of blubber missing) were present in all three cases in which grey seal DNA was detected. These defects mostly showed straight edges and angles, and grey seal DNA could not be demonstrated in these lesions (table 1). Given the DNA evidence from the smaller lesions present, five different types of skin wounds could be associated with grey seal interactions:

(1) The main mutilation: this comprised a skin and full thickness blubber defect. We set a minimum threshold of a 5 × 10 cm area of missing skin and blubber as representative of a grey seal bite wound and ignored smaller defects as these were interpreted as peck wounds made by birds.
(2) Head marks: one or multiple series of at least three repetitive, parallel puncture wounds anywhere on the head separated by a consistent distance of 0.5–2.0 cm (figure 1*b*).
(3) Tailstock marks: repetitive puncture wounds on the tailstock, present bilaterally, and running approximately dorsoventrally in two or more parallel lines (figure 1*d*,*f*).
(4) Flipper marks: a series of three or more repetitive incisions present on one or both of the flippers (figure 1*g*).
(5) Scratches: a series of three to five parallel running scratches anywhere on the body (figure 1*h*).

**Figure 2.**
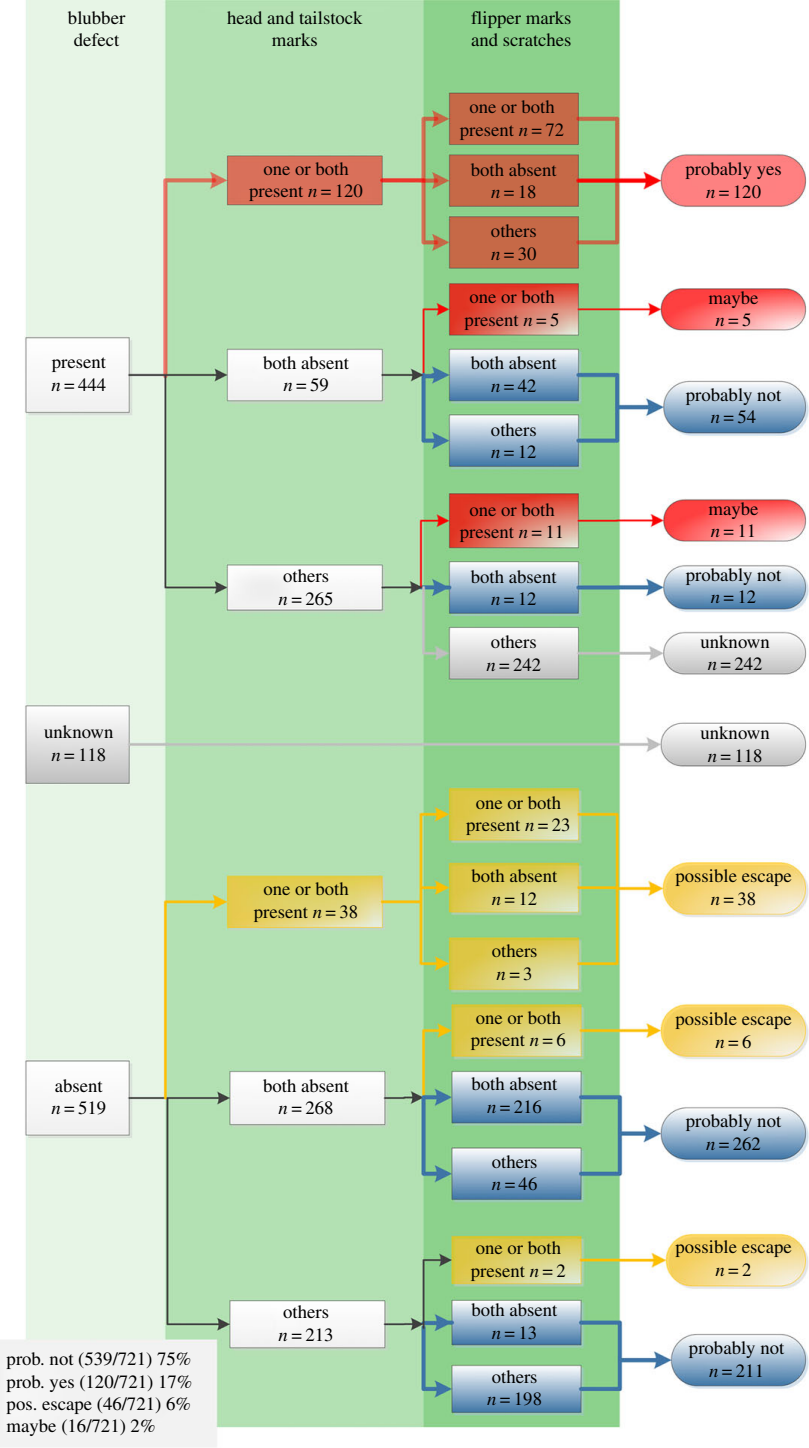
Decision tree showing number of cases that had presence, absence or ‘unknown’ for blubber defects, head and tailstock marks, and flipper marks and scratches, respectively. Others = absence of one characteristic, with the other characteristic ‘unknown’. ‘Probably yes’ = probable grey seal victim. ‘Maybe’ = possible grey seal victim. ‘Unknown’ = not possible to determine if grey seal victim. ‘Possible escape’ = victim that probably escaped from a grey seal attack. ‘Probably not’ = not a grey seal victim.

The presence or absence of lesions likely to be seal-related was determined in 721/1081 porpoises (figure 2); the remainder were too decomposed or not photographed in sufficient detail. Major blubber defects (main mutilation) were present in 444/721 (62%) porpoises. In 202 (46%) of these 444 cases, the presence or absence of marks on the tailstock, head, flippers or body could also be reliably assessed. In 120/202 (59%), head marks and/or tailstock marks were visible, and in 37 of the 120 porpoises both were present. In harbour porpoises that had no major blubber defects, head or tailstock marks occurred significantly less frequently (38/306, 12%; Fisher's exact test, *p* < 0.001). Flipper marks and/or scratches were found in 60% (95/158) of the porpoises that had head and/or tailstock marks (figure 2), whereas these occurred significantly less frequently in animals that had no head or tailstock marks (11/327, 3%; Fisher's exact test, *p* < 0.001). The significant concurrent incidence of a major blubber defect with one or more of the four types of marks prompts us to conclude that 120 animals were highly likely to have been victims of grey seal attacks (‘probably yes’ in figure 2).

Sixteen porpoises with a major blubber defect (2%) had no visible head or tailstock marks, yet did have flipper marks or scratches (*n* = 14), or both (*n* = 2). We consider these possible victims of grey seal attacks (‘maybe’ in figure 2: 2%). In 242 of the 444 (55%) porpoises with blubber defects, puncture wounds could not be reliably assessed and therefore the cause of the mutilations in these cases remains unknown. A final category of porpoises that had evidence of a seal encounter were those that lacked a blubber defect but did show marks on the head, tailstock, flippers or body. These animals may have been grabbed or bitten by a seal but probably escaped an immediate fatal seal attack (46/721, 6%: ‘possible escape’ in figure 2). In conclusion, based on the proposed assessment criteria, 25% (182/721) of the evaluated porpoises, the ‘probably yes’, ‘maybe’ and ‘possible escape’ categories (figure 2), had wounds attributable to a grey seal.

Gender and age distribution for the animals in the categories ‘probably yes’ and ‘probably not’ are shown in table 2. No significant difference was found for gender between the two groups (*χ*^2^ = 0.05, d.f. = 1, *p* = 0.824). Juveniles were significantly more likely to be victims of grey seal attacks than adults (*χ*^2^ = 8.0331, d.f. = 1, *p* = 0.005).

**Table 2.** Distribution over age and gender of the probable seal victims (‘probably yes’ category) and for ‘probably not’ category. For 110 out of 120 and 537 out of 539 cases, respectively, gender and age could still be assessed.

	male	female
probably yes		
adult	7	9
juvenile	53	39
neonate	1	1
probably not		
adult	51	79
juvenile	208	126
neonate	45	28

The distinction between attack wounds and scavenging defects was considered for the porpoises in the ‘probably yes’ category (figure 2). The cause of death could not be determined in 20 of the 120 available cases due to advanced decomposition or organ loss associated with the mutilation. In 90 of the remaining 100 animals, no definitive cause of death other than the presumed grey seal attack could be found. Four of the remaining 10 animals were emaciated and six may have died due to (an infectious) disease. Macroscopic haemorrhages were noted in 26 of the 90 animals for which no other cause of death could be determined. Eight of these were confirmed by histology.

The stomach contents were studied in 113 of the 120 porpoises in the ‘probably yes’ category. In 84 (74%) of these, prey remains were found in the stomach, whereas 29 (26%) had empty stomachs. Based on a detailed study of the stomach contents of grey seal victims, it was inferred that the nature of the wounding reflected their last meal [6]: porpoises with the main mutilation on the side of their body had eaten mainly demersal fish, whereas porpoises that had been mutilated in the throat region had eaten mainly pelagic, schooling fish.

The NCC could be reliably scored in 97/120 of the identified probable grey seal victims and in 271/539 harbour porpoises that did not show any signs of grey seal interaction (the ‘probably yes’ and ‘probably not’ categories, respectively: see figure 2). Animals in the ‘probably yes’ category had significantly lower NCC's than animals in the ‘probably not’ category (*p* < 0.001) and were thus nutritionally in a better condition.

These findings all indicate that the majority of 120 animals in the ‘probably yes’ category had been killed by grey seal predation and not scavenged post-mortem.

## 4. Discussion

The estimated frequency of harbour porpoise–grey seal encounters (25% of 721) includes the possible cases of grey seal attacks (‘maybe’ in figure 2: 2%) and animals that probably escaped an attack (6%). These findings suggest that grey seal attacks were the cause of death in at least 17% of the stranded animals. This is probably a conservative estimate as mutilated carcasses with an opened abdominal or thoracic cavity are likely to sink rapidly and decay, therefore going unrecorded. Moreover, animals that initially escaped an attack may have died later from the wounds inflicted. If dead stranded and autopsied harbour porpoises are representative of porpoise deaths in the region, then grey seal attacks (more than 17%) together with fisheries bycatch (approx. 20%), infectious disease (approx. 18%) and emaciation (approx. 14%) are the most important causes of death for harbour porpoises in the southeastern North Sea (Utrecht University 2009–2013, unpublished harbour porpoise autopsy results).

If grey seals benefit nutritionally from this inter-species interaction, then according to optimal foraging theory, they would preferentially target the most energy-rich parts of easily caught large prey [14]. Porpoise blubber fits this description of optimal diet better than most prey tissue. The porpoise population may suffer in ways other than loss of individuals as most of the mutilated animals were healthy and fat prior to the attack, suggesting that grey seals primarily target juvenile harbour porpoises that are in prime condition and so probably reduce recruitment to breeding age. For this reason, predation by grey seals may have significant cumulative effects on porpoise ecology as, under predation pressure, they may avoid profitable feeding grounds or adjust their diving behaviour in the presence of predators [15,16]. There is also increasing evidence that animals faced with a significant predation pressure may respond by losing weight to allow them to move faster, thereby increasing the probability of escaping attack [17–20]. Similar to the well-reported lethal aggression shown by bottlenose dolphins (*Tursiops truncatus*) [18], porpoises faced with the likelihood of seal predation may respond by becoming leaner and faster swimmers. However, weight loss makes a porpoise more prone to emaciation, another major cause of death for this species, and porpoise health may be impaired in a wider sense. As the smallest cetacean, the large surface-area-to-volume ratio means that porpoises lose relatively large amounts of body heat to their environment, forcing them to maintain high feeding rates. Both losing feeding time due to increased vigilance for predators and living leaner may pose a serious challenge for a harbour porpoise faced with a predation risk–starvation trade-off [18].

**Figure 3.**
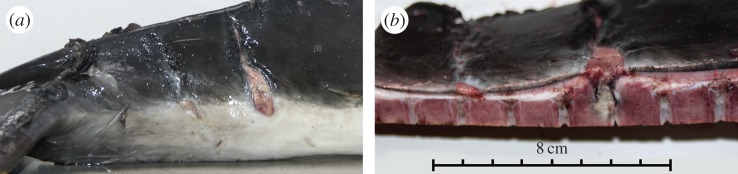
Example of a ‘possible escape’ case. Macroscopic photograph of an inflamed ‘tailstock mark’: (*a*) lateral view showing a skin wound similar in shape, location and size to ‘tailstock mark’ as shown in figure 1*d*,*f*, which shows partial healing; (*b*) cut section through the tailstock showing the same skin wound and inflammation extending into underlying tissue, new bone formation of the vertebrae and inflammation in the intervertebral disc.

Grey seal attacks on harbour porpoises are not always fatal, as shown by the animals in the ‘possible escape’ category (figure 2). Over 50% of the bite marks on these animals showed clear inflammation or healing, indicating that these animals had escaped an attack (25/46; figure 3). Such escapes would allow animals to learn to avoid grey seals, but at the costs mentioned above.

Another well-reported and frequent cause of sudden death in harbour porpoises is drowning due to fisheries by-catch. In these cases, post-mortem findings include all the characteristics of sudden death seen in grey seal attack victims except the bite wounds and associated haemorrhages. Without haemorrhages in the bite wounds, we cannot exclude the possibility that grey seals feed on porpoise carcasses bycaught in gill nets as they are known scavengers of fish entangled in such nets [14,21,22]. However, relatively few (*n* = 5, or 4%) of the ‘probable yes’ animals showed net marks on their skin, suggesting that if this phenomenon occurs, it happens infrequently. Still, it is tempting to speculate that harbour porpoises entangled in such nets may have triggered grey seals to turn from scavenging to attacking live animals. The first grey seal victim was found in 2003 [6], but without accurate information from earlier years it is not possible to determine when this behaviour first occurred. Increasing numbers of mutilated animals have been found from 2003 to 2013, but this trend parallels the increasing trend in the number of harbour porpoises stranded [6]. Certain prerequisites must be present for this behaviour to develop. These include sympatry of predator and prey, and possibly a high incidence of fisheries bycatch of the prey in static fishing nets to induce this behaviour.

Finally, many of the mutilated porpoises were found on Dutch shores used frequently by human bathers and surfers, and there would appear to be no *a priori* reason why humans may not be at risk from grey seal attacks.

## Supplementary Material

Figure S1.

## Supplementary Material

Figure S2.

## Supplementary Material

Basedata S3.

## Data accessibility

The data are available in a single Excel file (electronic supplement material, S3).
