# Supplementary figures and images for: Exposing the grey seal as a major predator of harbour porpoises
Source: Proc Biol Sci. 2015 Jan 7;282(1798):20142429. doi: 10.1098/rspb.2014.2429 (PMC4262184; doi:10.1098/rspb.2014.2429)

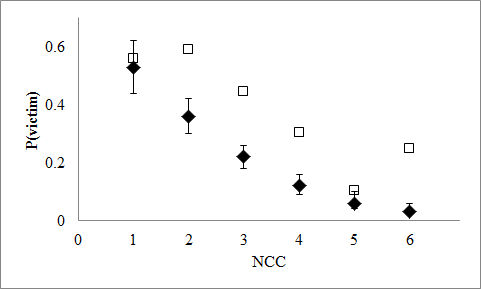

Supplement: Figure S1. [file rspb20142429supp1.tif]

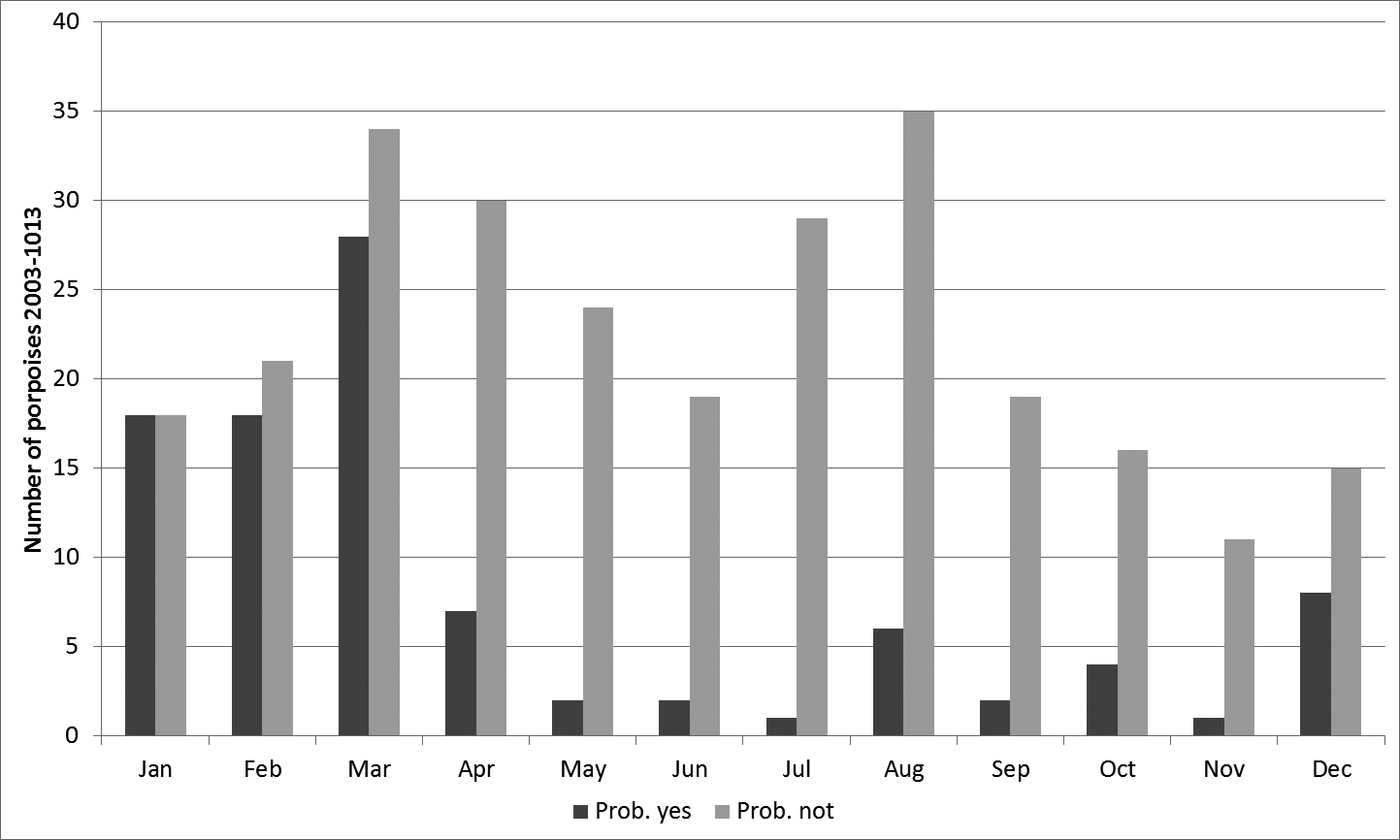

Supplement: Figure S2. [file rspb20142429supp2.tif]
